# World’s Columbian Dental College—1893

**Published:** 1893-12

**Authors:** J. M. Walker


### THE DENTAL REGISTER.

Vol. XLVII.] DECEMBER, 1893. [No. 12.

### Society Proceedings.

### World's Columbian Dental Congress—1893.

SCIENTIFIC WORK BY SECTIONS.

Reported for the Dental Register by Mrs. J. M. Walker.

THURSDAY, AUGUST 17TH.—FOURTH DAY.—GENERAL SESSION.

(*Continued from page* 568.)

The Meeting was called to order by Dr Shepard.

Dr. Walker, Chairman of the Executive Committee, introduced Dr. G. C. Daboll, of Paris, France, who was welcomed by the president as a special guest, representative of American dentists practicing in Europe.

Dr. Daboll briefly expressed his appreciation of the reception accorded him.

The report of the committee to promote the appointment of dental surgeons in the armies and the navies of the world was read by the Secretary-general.

*Mr. President and Gentlemen:*

The committee to promote the appointment of dental surgeons in the armies and navies of the world beg leave to report:

That opposition to such appointments came from the surgeons in the army and navy of the United States. The proposition to give the dental surgeon an equal grade with the surgeon was strongly opposed.

It was deemed the better course not to be too urgent at this time, but to send every year a copy of this request to the surgeon-general to remind him that the effort to place the dental surgeon on an equal grade with the surgeon has not been abandoned. Respectfully,

M. W. Foster, Chairman.

Dr. Walker then presented the official documents sent by the representation of Denmark. The first document was a testimonial from the Copenhagen Dental College authorizing Dr. J. L. Secher, dentist, of Copenhagen, to represent at the Congress the above named college and also the Dental Association of Denmark. Signed by the President, Ernst Warming.

The second testimonial was presented in the form of beautifully illuminated old English text. It was signed by the officials of the Copenhagen Dental Society.

Dr. J. M. Whitney, of Honolulu, read a paper entitled, "Among the Ancient Hawaiians." The paper was illustrated by a large collection of crania, maxillæ, etc.

To the thoughtful and conscientious dental student and practitioner there must constantly arise questions to which he may look in vain for satisfactory answers in his common surroundings.

Dr. Whitney said, that in view of the ravages of caries and other oral diseases to-day such questions naturally arise: Is this a necessary evil to which all mankind is subject? Or is it a result of the artificial life and varied foods to which our modern civilization binds us? Is dental irregularity due, as some claim, to the mixture of races, and if we could find a people homogeneous and simple would these conditions exist? Is it true that as mankind advances in the stage of being, the third molar is to become gradually eliminated? What is the normal position of adjacent teeth in relation to each other? What relation has the kind of food we use to the building up of dental structure? He considered that much light might be shed on these questions by the study of the crania of such a people as the native Hawaiians, who, till within a hundred years, lived isolated and unknown to the great world, therefore their habits were simple, and their wants and opportunities few, and whose modes of burial were such that it is possible, with comparative ease, to obtain some knowledge of their primitive conditions. It would be difficult, indeed, for most of us to find living subjects, in any number, meeting these conditions. But fortunately the bony structures are preserved long after the owners of them have passed beyond mortal ken, and if we can obtain the crania especially of people, who, living upon the earth ages ago, when wants were few and means of supplying them were correspondingly limited, our study of their dental conditions will certainly be interesting and ought to be instructive. I consider myself especially fortunate in living in a land where both of these requirements are met.

Dr. Whitney then sketched briefly the history of these islands and the mode of living, diet, burial customs, etc., of the ancient people. Of those buried in the sands of the seashore very little now remains of the thousands of human beings whose bones once lay bleaching under the tropical sun. The natives have broken and demolished the skulls, jealous of their removal, while the South Sea Islanders have removed the teeth for their barbaric necklaces. But, fortunately for the modern scientific investigator the superstition of the more ancient Hawaiians led them to deposit their dead in the most remote and inaccessible places, such as the old lava caves, where no wind or moisture ever penetrates, the remains being still as perfectly preserved as in our modern cabinets. These ancient sepulchers are guarded with most jealous care, and it is very difficult to obtain access to them. Dr. Whitney gave a most interesting account of the difficulties encountered in his finally successful search for one of these ancient caves, whence he returned with well-filled bags of the crania of this most primitive people.

He said: We have been taught that primitive peoples, living in simple conditions, were, in a great measure, free from dental caries as we see it in the mouths of our patients, and that many of the forms of dental disease with which we have to contend were with them wholly unknown. This seems to be a mistaken teaching as far as may be learned from these records. An exceptional opportunity of becoming acquainted with the crania of the ancient people of these islands, during the twentyfour years of my residence there, has convinced me that both in the case of those buried in the caves, and of those more recently in the sand, not more than twenty-five per cent. have been free from caries, irregularity or disease. Indeed, I think I have discovered every form of dental disease known to our practice; dental caries in all its many types, necrosis of the teeth, erosion, alveolar abscess, pyorrhœa alveolaris, disease of the antrum of Highmore, necrosis of the maxillæ, anchylosis of the jaw, salivary calculus, etc.

Perhaps, next to dental caries, the greatest source of oral disorders among these people was the irregularity of the third molar, often producing in them as serious consequences as with us of the present time, while its failure to erupt was nearly, or quite as common as we find it in our daily practice. So that we cannot argue from these remains, at least, that the coming man is to be deprived of this useful organ. The children of the natives of the present generation, as shown by actual examination, have but little better teeth than their white school-fellows. Their fathers and mothers may have better teeth than the children, but it would be an exception if they had not been to the government physician and had one or more teeth removed for relief from odontalgia, while the grandparents, the old men and women whom I found when I first went to the islands, had teeth proximating those found in the old caves, though not as good.

I lay much of this great change to the many forms of disease that have weakened their constitutions; to fine flour that has become a part of their diet, and eaten in the form of crackers, or hard bread, clings to the teeth; to the many acid fruits, such as tamarinds, guavas, limes, etc., to which they have constant access, and to spending their childhood and youth in the school-room instead of wading and swimming in the warm sea; eating raw fish and shell-fish which they have caught, chewing sugar-cane, and stripping off with their teeth the fibrous covering of the cocoanut.

Dr. Whitney stated, after reading his paper, that he had many specimens of crania in which the third molar was not erupted, and he had found that it was as frequent among the ancient Hawaiians not to erupt that tooth as among his present patients.

DISCUSSION.

In the consideration of this paper Dr. Peirce, of Philadelphia, called attention to the width and large ramus of the jaw, and yet, with all this space and weight the third molar entirely lacking; also to the tendency in ancient skulls of a secondary development of the process, replacing, in a measure, lost teeth—in one case a sharp incisive process where the upper bicuspid, cuspid, and molar had been lost, the lower jaw retaining the teeth.

We find in the large collection of the Academy of Natural Sciences a great many skulls where the teeth having been lost for many months the friction of the adjacent parts upon the processes has caused a secondary developing process, which, to an extent, has taken the place of the teeth.

This discovery belongs to Dr. Harrison Allen, of Philadelphia, who was the first one to direct attention scientifically to that fact. In modern jaws, where the teeth are all extracted by dentists, and plates are introduced making pressure over the whole jaw, we do not see such an appearance as in these jaws, the pressure and friction being more uniform. The fact that we find evidences of the presence of all diseases that are found at the present time is a very interesting one, showing that whether pyorrhœa, abscess, diseased antrum, or decay, they all existed as they exist at the present time. But how much may be due to the fact that these were an isolated people, intermarrying for several hundred or thousand years together, and cut off entirely from the outside world, is a question to be taken into consideration.

There being no further discussion of the paper, Dr. Hayhurst, of New Jersey, then read a brief abstract of "The History of Dentistry in the United States," compiled by him from material gathered by Committees No. 8 and 18, of the Columbian Dental Congress.

The chairman announced that by special vote five minutes would be allowed a veteran in dentistry to deliver a few remarks, and introduced Dr. Corydon Palmer, who said :

I belong to that period when the advance in modern dentistry began—more than fifty-five years ago, in 1839. Tuition could not be had by a young man except in the large cities and for a price from $300 to $1,000, and no young man was allowed to go into an operating room to see an operation.

During a period of fifty years I have learned things by trying them. If I know anything it is because I have tried it.

I have three ideas I wish to speak of:

First. I would urge you not to practice the extraction of deciduous teeth in children. It is cruel. It produces irregularity and deformity in future life.

Second. Be careful not to overtask young patients, girls and nervous persons. Don't keep them in the chair too long. I have seen sensitive patients much injured by it.

Third. As to the use of arsenic : I began to use it in 1859, but soon learned its disastrous effects. Use it as you will, it is still arsenic. It belongs down below, and if you use it you will have a nightmare that will follow you to eternity.

The President then made a number of announcements relating to the Congress, after which the meeting adjourned to Friday, August 18th, at 12 M.

---

### WORK OF THE SECTIONS, THURSDAY, AUGUST 17.

---

### SECTION 1.—ANATOMY AND HISTOLOGY.

Section called to order by Dr. Sudduth in the absence of the Chairman, Dr. Andrews.

The chairman called for the discussion of last night's papers. There being no remarks the section proceeded with its regular paper entitled "The Pedigree of the Central Incisor," by Dr. A. H. Thompson, of Topeka, Kansas.

The condition of the upper central incisor in man is something unique, in that while all the other teeth are reduced in form and degraded in specialization, more or less, the central has not only preserved its special form and maintained the practice of its function, but has indeed advanced somewhat, being rather more highly specialized in man, as an effective cutting instrument, than in some of the lower forms.

The lateral incisor, while highly developed in most individuals, is frequently reduced in form, and sometimes is totally absent. This is not infrequently the case with the cuspid. The bicuspids, molars, and lower incisors also show the effects of degenerative modification; the central incisor alone maintains a high degree of completeness and specialization in man, which fact makes a study of its genetic evolution a matter of peculiar interest.

The evolution of the organs or apparatus designed to effect the function of dividing or cutting food was traced by Dr. Thompson, beginning very low in the scale of life. The cephalopods, the mandibles and the pinchers, of insects and crustaceans; the saw-like mandibles of the leech, etc. The lowest form in which teeth, with any approach to the true incisor form are found, is the sea urchin, *Echinus*, which has five incisors arranged around a central point in the remarkable apparatus called "Aristotle's Lantern." They are used with great force to cut shells and rocks. These teeth are set in true alveoli and are worked by powerful muscles.

In the fishes there are no incisors, properly so-called.

None of the reptiles have cutting-teeth proper.

The beaks of turtles are analagous to incisors, but are not homologous with them; the same may be said of the bills and beaks of birds.

Most of the lower mammals are deficient in regard to incisors, usually having teeth on the sides of the jaws for grinding purposes only. The Kangaroo has large cutting incisors, as it is an exclusive vegetable-feeder.

In the rodents we find the central incisors developed wonderfully, into the long, continually-growing implements used for cutting.

In the herbivora all of the incisors and also the canines are highly developed for cutting purposes. A curious exception is noted in the ruminants, the most of which have no incisors in the upper jaw.

In the Elephant and Mastodon the central incisors are developed into long tusks, which are employed as effective digging implements and piercing weapons.

With all these, however, man has little relationship.

The order of insectivora presents many remarkable forms of the central incisor. In *sorex* the large upper incisors appear bifurcate, from the great development of the posterior talon. This talon is often repeated on the lateral incisor in man, where a lingual cingulum is not uncommon. This is, perhaps, the precursor of the large interspace.

The Hedgehog has large centrals, sometimes with a large interspace, which is often present in man, and is so frequently a matter of heredity.

Some of the curious forms exhibited in the insectivorous series are passed over in the cheiroptera, and reappear in the quadrumana, and occasionally in man.

In approaching the quadrumana, the highest branch of animal life related to man by collateral descent from a common ancestor, the lowest family of the order is that of the Lemurs, which present considerable variation in the form of the central incisor.

One of the lowest, and one closely related to the insectivora, is the little flying Galeopithecus, in which the lower centrals present the form of a comb, produced by the deeper extension of the marginal notches into the crown. These are analogous to those on the edges of the human incisor, but the notches are more numerous and deeper. This tendency to division is recalled by the tubercles upon the edges of the incisors of man when first erupted. The longitudinal ridges, which lead away from the tubercles, are also suggestive of this primitive division of the crown, as an embryonic record of the history of its evolution.

The lowest monkeys, the platyrrhines or wide nosed, the American species, are closely related to the lemurs in many respects, notably in having the third premolar. The central incisor begins to approach the final form in the higher groups, but is still somewhat aberrant. In the platyrrhines it is still more or less of a scoop-shape, resembling the lemurs. The scoop-shape is often recalled in man, with the characteristic curve and thin edge.

In the catyrrhines (narrow-nosed), the Old World monkeys, the centrals are much wider and larger than the laterals, but nearer the shape found in man, even in the lower form. In some of the baboons there is more or less of a basal ridge, which is often greatly developed on the lingual face. This lingual ridge is often recalled in man, but not on the labial face.

In all the anthropomorpha the centrals are much larger than the laterals, are more vertical in the lower forms, and assume the final shape presented in man. There is little real difference in the form of the central incisors between the higher apes and man, except in the size and quality of structure and in the comparative size of the centrals and laterals. In man the extreme disproportion manifested in the apes is much reduced, *i. e.*, the centrals are smaller, and the laterals are larger.

In the Orangs the centrals are of great size, are twice the size of the laterals and have the basal ridge. This extreme size is recalled in man by those examples which we see of excessively large centrals. Tomes says these teeth are similar to those of man, but larger. In the Chimpanzee the centrals are very much reduced from the size in the Orang, and approach the proportions of the same teeth in man, but with a prominent basal ridge as in the Orang.

In the Gorilla the centrals are still nearer the final shape, as in man. They are of the same form and proportions, but are larger and of coarser structure than in man, as are all the teeth of the greatest ape. There is little real difference between the central of the higher apes and man, for it assumes the final shape long before man is reached, and must have taken on this form before the differentiation which separated the human branch from the quadrumanous.

As showing the persistence of type and the singularity of the survival of this tooth in such perfection, we find that the central incisor of man has lost nothing in the course of the evolution of the species, and yet it is little elevated above that of the apes. In fact, the general structure of the denture of man is degraded and primitive. The central has lost nothing in the process of the destructive evolution of the species, for while the other teeth have been degraded, it has maintained its own, and is really superior to the others in the retention of the perfection of its functional specialization.

DISCUSSION.

In the discussion of this paper, Dr. Eben M. Flagg, Asuncion, Paraguay, said: I would like to ask if the doctor thinks that the central incisor in man has reached its complete culmination, or whether, as Dr. Bonwill says, that it started and has since degenerated?

Dr. Thompson: We can hardly expect that the cutting function will be much increased. The chances are that with the rest of the denture it will follow rather in the line of degradation than advancement. Many of the teeth of the human denture are much degraded, and some are on the road to entire disappearance. The third molar is very erratic, and I think is being lost. The next is the lateral incisor which also will probably disappear. The central incisor is really unique, because it does not indicate so much degradation as any of the other teeth.

Dr. Sudduth: In what direction does Dr. Thompson think this retrogression will present itself, whether as to crown or root, or in what form will it occur?

Dr. Thompson: There is only one way to answer that, and that is, that the lessening function will necessarily lessen the impulse of development in a particular organ. It is a question whether, in the higher development of the species, the teeth will be entirely reduced and destroyed. I imagine they will be preserved for other functions through the law of collateral variation. I am not inclined to be as confident now of the entire loss of the teeth as I was at one time. The collateral variation will tend to the preservation of teeth for other functions than mastication.

Dr. Sudduth: I have noticed in studying collections of teeth in relation to dental anatomy, that there seems to-day a larger proportion of short-rooted centrals than there used to be. It may be that I have, within the last five years, come across a larger proportion accidentally, but it seems to me that in handling many thousand teeth each year, as I do now, that I have been struck with the point that very many central incisors had short, stunted roots. The query has come to my mind whether that is not a part of the necessary process of retrogression, which comes from the lack of the use of the teeth in this country, where we use so little hard food, and our food is prepared in a soft form. That is why I ask the question.

Dr. Abbott: In the skulls presented this morning in the general hall there is a chance for study and observation. I have a theory in reference to the stunted condition of the teeth, that it is due to a specific trouble, or disease in the system. You know very well that the papillæ of teeth are affected very easily by disease, and that the disease may be so obscure as to not be noticeable to the individual or any other person, and still you can find out that such and such a thing produces the disease, and if we would turn our attention to that one direction, to the history of the individual from generation to generation, we would find that one of his ancestors would have presented sufficient cause in his condition for us to believe that the disease was transmitted to the individual. I have felt that there was an opportunity for a great deal of real study in that direction. That nature herself, for want of use is to dispose of the teeth, in part or in whole, I cannot conceive, for I have never seen anything to make me think so.

Dr. Thompson: In regard to the shortness of roots of which Dr. Sudduth spoke, I think that is a matter of temperament, or of ethnical peculiarities, because there is no doubt that there are distinct racial types of teeth, if we could diagnose them, just as there are of other parts of the body. That follows in the line of evolution and change. Of course the changes that are brought about in the evolution of the species are not to be noticed in the lifetime of any individual.

Dr. Eben M. Flagg, of Asuncion, Paraguay, read a paper entitled, "The Human Temperament in Relation to the Human Tooth."

Every person possessing the faculty of observation must have noticed the remarkable differences that characterize individual members of the human family. The term that has been used to express these differences in the physical and mental constitution of individuals is temperament. No matter how much individuals may have differed as to the way this study should be pursued, all have tacitly agreed that the word is appropriate to express the ideas sought to be conveyed. Various theories have been advanced to account for those different mental and physical qualities under consideration, which inhere to man and are not possessed by any other animal, and it has been sought to prove that they are but the results of his environment; that they are produced by his surroundings, such as soil, climate, locality, habits of life, etc., upon his physical and mental frame. I feel impelled to take an agnostic position, to say candidly, I do not know, but have much hope that future investigations will bring us nearer to the cause. If I may be permitted to express an opinion, I would suggest that the temperament seems to be due rather to mental than to physical causes. Where both are given free play it seems to be in accordance with the law of nature that mind should predominate. In speaking of mind, I mean the function of the brain.

The writer then reviewed his classification of the temperaments, published ten years ago (*Cosmos*, Feb., 1884), and Prof. J. Foster Flagg's tabulation of the binary temperaments, citing illustrations of the various combinations met with as exemplified in some notable individuals, all tending to illustrate the importance of the study, to the scientific dentist, and to prove that we cannot afford to ignore it.

Dr. Thompson: The paper struck me as being something which needed expression in our college work. I have had occasion to regret that there was nothing explicit in our literature which we could use in our instruction to the student in regard to this particular thing. It is very necessary that the practitioner should be able to diagnose the different temperaments, particularly in regard to the making of artificial teeth for edentulous patients. It is very important for the young practitioner to distinguish at least one temperament which predominates in the individual.

The temperaments are manifest to us in Europeans more than in other races on account of our greater familiarity with them. Temperament exists among other races and influences the individuals just as much as in the European races. It exists also in animals. Some horses are more nervous than others, and have a different organization. That brings us back to the idea of the men who first described temperament, that it may be organic and not ethnical after all. The question is still very much confused and in want of light, and any contribution to it is very valuable.

Dr. Sudduth: The subject of temperament is one that has interested me very much, and as Dr. Thompson says, it should be taught in our colleges. I have always had an interest in the study of physiognomy. In the face we have depicted the attributes of mind, and to a certain extent, the innermost thoughts of the individual; in addition to that, dentally, the teeth are also types of temperament. There is a correlation between the physiognomy and the teeth; a marked analogy, which can be borne out, certainly exists. Given the teeth of an individual, one who is well up in those studies can tell you what the physiognomy is, or given the physiognomy, can tell you what will be the teeth. There is a great correlation between these two features of the anatomy.

There is no doubt but that temperament has very much to do, as Dr. Flagg has indicated, with the intensity of inflammation. That is a point that has been described and is well known. For instance, a sanguine temperament is one in which we have all inflammatory processes taking on a very acute form. Not only does the sanguine temperament tend towards acute inflammations, but if the remedy is applied in the right way the inflammations respond readily. The lymphatic temperament is, to a more or less extent, a negative temperament. It does nothing in a very vigorous way, either as to getting sick or getting well. It is a slow, sluggish and undesirable temperament to possess or to treat.

Dr. Flagg: Although I have not been able to make as full a presentation of the study as I would like, I think if my paper will serve as a basis to interest scientific men in the subject, that I shall feel fully compensated for any study I have made in this direction.

Adjourned.

SECTION 2.—ETIOLOGY, PATHOLOGY AND BACTERIOLOGY.

The Chairman, Dr. Black, called the meeting to order.

Dr. I. P. Wilson read his paper entitled, "Pathological Conditions of the Air-Cavities of the Cranium Resulting from Dental Lesion."

Diseased conditions of the air-cavities of the cranium are of frequent occurrence, and very often of uncertain origin. Nasal catarrh is usually the outward manifestation of these pathological conditions, the nose being the only outlet for the secretions from these cavities.

An exploration of the nasal fossæ will reveal the fact that more than a score of chambers or cells open into this convoluted passage.

We find that all these cavities communicate freely with the nostrils. We also find that one continuous stretch of mucous membrane covers all of these uneven surfaces.

A pathological condition of any one of these parts must, therefore, necessarily endanger the health of other parts contiguous therewith. These diseased conditions are usually known by the ambiguous name of "nasal catarrh." The seat of the disease is too frequently overlooked, and treatment is directed to the *effect*, and not to the *cause* of the malady.

The object of this paper is to call attention to the fact that pathological conditions of any or all of the air-cavities of the cranium not unfrequently have their origin in dental lesion.

Remembering that the apices of the roots of the first and second superior molars are separated from the maxillary sinus only by thin plates of bone, and that in some instances these roots penetrate the floor of the antrum, with nothing but the soft tissues of the mucous lining covering them, we can readily understand how easily an outlet can be gained for putrescent matter and gases through the antral cavity and into the nose.

The writer then traced the course of the disease, beginning with an aching tooth and the formation of an abscess at the root of one of these teeth, which was followed step by step and illustrated by a detailed report of a typical case, with the treatment, until a normal condition was restored.

In conclusion he said: Medical writers upon pathological conditions of the ethmoid cells, and the other air-cavities of the cranium, including the antrum of Highmore, rarely ever mention the teeth as even a possible cause of disease in these cavities, or as in any way affecting the general health.

Diseases of the human teeth, and the dire consequences that so often follow, not only in the direction where equally grave results so often manifest themselves, involving the health and happiness of thousands of human beings, should be a matter of the greatest concern to both the medical and the dental professions.

DISCUSSION.

Dr. Frank Abbott, New York: I have had more or less experience with the subject treated, and have met with much the same experience as Dr. Wilson has stated. I have arrived at several conclusions: First, that medical men, as a rule, do not take into account the conditions that present themselves on account of the diseases of the teeth. They would, for instance, treat for years a case of nasal catarrh, and the patient might die on their hands and they never ascertain *the cause* of the trouble.

The second conclusion is: That in all cases of disease of the antrum it is almost and entirely sufficient to first remove the cause of the trouble and then keep the parts clean, and unless that idea of cleanliness is followed out it makes no difference what is used in the way of antiseptics or anything else. The parts must be kept clean, and if that is done it is the best antiseptic treatment that can be thought of. This may be done with warm water alone, or with a little salt added.

Dr. Ingersoll, Keokuk, Ia.: The subject illustrates one fact, and that is the influence of small causes in producing serious results.

I do not think medical men or dentists have sufficiently considered the importance of the smallest causes when long continued. Prolong any irritation, and it becomes a very serious irritation. Let a drop of water fall on a man's head, and no harm is done, but let it continue for days, and the drops of water will drive the man insane. Let a mote be thrown into the eye, at first it is scarcely perceptible, but it may produce the most serious results. A little pulp is left in a canal, it may go for years without giving trouble, but subsequently the trouble comes.

The discussion was continued, and cases cited by Drs. G. V. Black, E. S. Chisholm, A. O. Rawls and G. Vargas Paredes, Colombia, S. A.

The subject was then passed, and the first assigned for discussion was then taken up. The subject was: "Can Apical Pericementitis Occur in Connection with Roots which have been Perfectly Sterilized and Filled; and if so, Under What Circumstances?"

Dr. A. O. Rawls, Lexington, Ky., opened the discussion, and said, that to sterilize a tooth perfectly it was necessary to go beyond the ordinary root-canal clear through the cementum. The speaker did not believe that there was any anastomosis between the tubuli of the dentine and the stellate structure of the cementum. If you can fill the canal up to the extremity, then, in all probability, you have done all that is possible to do to prevent pericementitis.

Dr. Wilson said, that there was one other cause of pericementitis after the root had been thoroughly sterilized and filled. When we examine the histological structure of a tooth, we find a very small portion of the apex that is all composed of cementum; that small portion has, without any doubt, a direct communication with the pulp. Now, if we apply arsenic to the pulp of the tooth more than once, we are in great danger of devitalizing the pulp, not only throughout the main body but down to the very apex of the root. We have then destroyed that part of the pulp that is in direct communication with the cementum, and thereby destroyed the vitality of the cementum at that point. It then becomes a foreign body. I always feel afraid when that is done that I have destroyed the life of this apical portion of the cementum, and that no matter how thoroughly the canal is sterilized and the tubuli and the canal filled, that there is still a little foreign substance at the end of the root—the source of that uneasy sensation so often complained of.

Dr. Fernandez, Chicago, prefers the mechanical modes of pulp extirpation. He said: I think the main cause of pericementitis is the poison used in destroying the pulp. You have the circulation destroyed when the poison gets there—the capillary circulation is destroyed. The obstructed functions of those parts is, to me, where the trouble lies.

The second topic assigned to SECTION 2, "To what Extent is Interrupted Primary Dentition an Etiological Factor in the Diseases Incident thereto, More Especially Pulmonary, Digestive and Intestinal Disorders," was passed, no one offering to discuss it.

The Section adjourned to meet Friday, at 2 P. M.

### SECTION 3.—CHEMISTRY AND METALLURGY.

This Section held no meeting.

### SECTION 4.—THERAPEUTICS AND MATERIA MEDICA.

Dr. F. J. S. Gorgas, in the chair.

Dr. Thomas Fillebrown, Boston, Mass., read a paper as follows: "A New Apparatus for Maintaining Anæsthesia without a Face-Piece, and with the Mouth-Open."

The necessity of repeatedly re-anæsthetizing the patient has always been a great and serious hinderance to the progress and success of surgical operations within and about the mouth and throat. My own experience in operating upon the palate and lips led me to consider the possibility of maintaining the narcosis without interfering with the operation. I am pleased to announce the accomplishment of this, in the conception of an apparatus to accomplish the much desired object of anæsthetizing without sponge, towel or face-piece.

My apparatus consists of a bellows, connected by rubber tubing with the long tube of a twelve-ounce wash bottle, with a stop-cock intervening to regulate the flow of air. From the bottle extends a half-inch rubber tube to the patient. The bottle is filled one-third full of ether. The bellows is inflated and the stop-cock opened so as to allow the air to bubble up freely through the ether, and to become saturated with ether vapor. The etherized air is then discharged through the second tube a few inches from the patient's face.

This application of ether will maintain complete anæsthesia for any length of time, and not interfere, in the least, with any operation in or about the mouth; nor will the surplus vapor discharged into the air sensibly affect either the operator or the assistants. I have made a still further modification of the instrument, and adapted it to the induction of initial anæsthesia, and its maintenance with face-piece for use in general surgery. I attach to the discharge tube of the apparatus, above described, a double-valve face-piece, such as is used for the administration of nitrous-oxide, and a double-end gas bag for a reservoir. By this means the patient is insured an abundance of pure anæsthetic atmosphere for each inhalation. Narcosis is induced with absolute freedom from any of the disagreeable symptoms usually experienced when etherization is accomplished, by simply raising the tube in the bottle until it is entirely free from the ether, filling the bag in commencing the inhalation. In this air there will be but a very little ether. Then gradually slide the tube down into the bottle, and as it approaches the ether the strength is increased; after one-fourth of a minute the patient can breathe the full strength.

If the mouth is to be operated on, when anæsthesia is complete, disconnect the bag and face-piece and proceed as before described.

In reply to questions asked, Dr. Fillebrown said, that while anæsthesia was not produced as quickly as by the old methods, there was entire freedom from coughing, and very much less emesis.

Dr. J. E. Cravens: I will say to the gentlemen that if they will have the anæsthetic agent, chloroform, ether, or gas, inhaled through the nose, they will have none of the trouble they mention.

Dr. Caracatsanis being present, his paper on "Treatment of Alveolar Pyorrhœa," read and passed without discussion at a previous session, (see page 551), was re-read by the author.

DISCUSSION.

Dr. James Truman, Philadelphia: I cannot quite reconcile this treatment with my ideas of the disease. The gentleman, as I understand it, takes the position that he must scarify the gums. To my mind the gums are not the origin of that disease. What is its source? If I understand it, it originates in the pericementum. Pyogenic germs generate there and produce the irritation in the primary stages; as these progress, necessarily the pockets deepen, and the gum is left almost intact. Now, why scarify the gums?

If the position I take be the correct one, that it is owing to the microbic influence in the pocket that this disease occurs, then the first thing to do is exactly what this gentleman did: use a powerful agent such as mercuric chloride in the proper solution. That is the beginning; then he applies iodine and aconite. Now, I cannot comprehend exactly what effect iodine and aconite will have for the checking of this pathological condition. Aconite, if I understand it, is to obtund the nerves more particularly; it paralyzes the nerves, and prevents by their action the influx of blood to a particular part. Iodine is simply an irritant when used in that position, and as far as that goes it has its usefulness; but to my mind, while it may be good, it does not meet the conditions present. We want something more than this. The antiseptic that is used, or may be used, whether it be mercuric chloride, hydronaphthol, phenol, or any other agent, must necessarily be used continuously. I think the great mistake made by operators in this direction is that they do not meet the conditions for the future; they consider the case cured when they leave it, after perhaps two, three, or six weeks. But that is simply the beginning; if the patient is to be treated properly, it must be continued through the balance of the life of that patient. I never would allow a patient to go from my office and tell him the case was cured without giving him the proper remedies to use, and proper antiseptics to keep the pathogenic germs from producing further decomposition in the future. Unless explained properly, the patient will only return in a few weeks with the case just as bad as in the beginning.

Dr. Cravens gave his method of treatment, as follows:

He said, I have had from one to five cases of pyorrhœa alveolaris almost every day, either in the first, second or third stage, and I have not had a failure in the case of a single tooth or pocket that I have treated.

The first step is to scrape the root of the tooth. I do that after anæsthetizing locally, so that it is possible to scrape the root painlessly; then I wash out the pocket with the hot water to get rid of everything that is loose in it. I then apply dilute sulphuric acid, one part of the sulphuric acid to ten parts of water, using the common commercial sulphuric acid. I apply it in two ways,—with a syringe, or a common quill toothpick; a very useful thing where you wish to use sulphuric acid or nitrate of silver. It is very flexible, and is not affected by any of the acids you use. I want to fill the pocket with this sulphuric acid, that is all; if it escapes in the mouth it will do no harm beyond perhaps etching the teeth a little bit.

That is the end of the first sitting. The sulphuric acid will occasion pain in applying it that way, but if the operation of scraping has not been very long, you will find that the effect of the cocaine will hold over, so that the sulphuric acid will not occasion any pain. I don't care about the strength you use, but if you think the effect of the cocaine has subsided sufficiently to allow the patient to feel the effect of the acid, apply the cocaine again.

The second sitting is not a surgical one; the first one should complete the surgical part. I begin with the hot water, and thoroughly wash the pockets as many as four or five times with an ordinary plunger syringe, forcing it in with just as strong a pressure as I can make; then I follow with a ten per-cent. solution of nitrate of silver. That solution is not caustic, because you cannot get the pockets totally dry. You leave some of the water there, so that when you put your silver solution in, it is instantly diluted. I use just enough to fill the pockets, because if you have an excess and there are any gold fillings in the mouth, it will discolor them; or if you use a great deal, you might very seriously injure the surface of the teeth. One application of the nitrate of silver ought to be sufficient. Then I discharge the patient without any further treatment at that sitting. For the third: I usually allow four days between each sitting, though the period might be increased to six. I wash out with the hot water, and for the nitrate of silver I substitute bromo-chloralum, which you can purchase at any drug store; I believe it is used for a general disinfectant, embalming fluid, and all sorts of things. It is a powerful astringent. I use it with full strength and in the same way, either with a syringe or with a quill.

There should be but three sittings. If there is pus evident at the third sitting, you will have to scrape the tooth again, because you have not done it properly in the beginning. But if it is done properly there will be no pus after the first sitting. I then have the patients come once or twice every one, two, or three weeks, to have the pockets washed out with warm water, and give them a douche of the bromo-chloralum that is diluted, perhaps one to five or six, and I take occasion to examine the case to see if it is all right; but there have been no cases in which there was a recurrence of pus.

Dr. W. J. Barton, Paris, Texas: The absolute similarity of the treatment is not so important, so that the surgical operation is thorough, so that the treatment is thoroughly antiseptic and cleansing, and the pockets are kept cleansed for the time. That is a very excellent mode of treatment; I like it very much. I have had in my practice cases of seemingly successful cure, and I proceeded in about the same course of treatment.

Dr. Harroun also gave his testimony to the success of Dr. Craven's method, in his own practice.

Dr. Hart, California: The treatment I have pursued has been very simple. I take it for granted the disease is a disease of the periosteum of the tooth, and the periosteum is dead so far as the disease has advanced. It will go almost to the apex of the tooth, and perhaps three-quarters of the way around.

I begin with a treatment of bichloride of chinoidin. I only use this once, and the next time my patient comes I make a band of No. 60 gold, that goes clear down underneath the gum as far as the pocket has gone, and put it on, encircling the tooth on the other side also, and cement that firmly in position, and I have the best kind of success. The disease seemed to stop after passing that band of pure gold underneath the gum, of course, removing all calculus that might be present, but sometimes there is not any.

To allay inflammation I use europhen, in a saturated solution, in campho-phenique. I use a mouth-wash of hydronaphthol, and it is a splendid mouth-wash.

Dr. A. O. Rawls, Lexington, Ky.: I claim that the original cause of this condition is either inherited or acquired, and a result of the use of mercury. Very often inherited, too, from two or three generations. All you can do by your treatment is to arrest it for the time being. There is no special medicament that is better than a perfect surgical operation, but I apprehend the majority of surgical operations in these cases do not go far enough; they only reach the root of the tooth. In most of these operations you are compelled to go beyond the root of the tooth. The process, as a rule, is broken down, the membrane is entirely swept away, and unless you reach the farthest limit you will have no cure. As to the treatment—once or twice being successful in all cases, I do not think it is so. I don't think any one medicament or substance or two or three treatments will cause a successful issue in all cases. Very often cases which are called typical cases of pyorrhœa you will find are nothing in the world but the result of uncleanliness and negligence, and the accumulation of tartar around the teeth.

Dr. Taylor: I should say from this diversity of opinion that there were two kinds of pyorrhœa alveolaris: the one we meet in the office, and the one we meet in conventions. The cases we meet in conventions are very easily treated, but those we have to deal with in practice are very difficult, and cause us much trouble and tribulation.

Dr. J. Y. Crawford, Nashville, Tenn.: We have one distinguished member of the dental profession in this country who has made a statement in reference to this condition and its management, and I regard that statement as sufficient to settle at once the character of this disease: that it is a local manifestation, depending upon constitutional conditions in some cases, and local conditions in others. This gentleman made the statement that it was a self-evident disease; that he challenged the world to find a single solitary case that did not get well when the teeth were all taken out. That was Dr. H. W. Morgan, of Nashville, Tenn., and to my mind it was the most philosophic expression I have ever heard made upon the subject.

Now, I want to say in the treatment of this disease, as in other forms of diseases of the mouth, two wrongs have existed; one is that in the management of them too vigorous surgery, is in many cases applied, and another is too much medication. When you get the case in a condition of improvement and comfort, let it alone. Avoid all medication.

When you get the effect of your treatment, and a condition of comfort established, let it alone unless some condition arises that demands attention. What medicines you do use let them be intelligently selected; don't put in a septic cavity or during the septic condition a counter-irritant.

Dr. Hoff, Ann Arbor, Mich.: I do not believe that disease is always or that it is often of local origin. I believe that it is systemic, and I think that you will find in these very bad cases of pyorrhœa alveolaris, that all the patients are troubled with diseases, especially, of the eliminating organs of the body, the kidneys, the liver, and so on. I think where we make the mistake is, in not taking into consideration the systemic condition of the patient; and I think when we get fully to understand this disease, we will find that it has a closer relation to the system and disturbances of the body than we now realize.

Dr. Cravens, in reply to the preceding speakers, said that the best refutation he could make would be to invite the gentlemen to come to his office and make an inspection of the cases referred to.

The paper of Dr. Poinsot, of Paris, France, upon the subject of "Extraction of the Pulps from Teeth in a Calcified State by

Trephining," was read by the Chairman, of which this is an abstract:

Physiology teaches us that the tooth receives its nourishment from two different sources: The external life of the tooth, through the peridental ligament, and the internal life through the dental pulp, the function of which consists in securing the calcification of the organ in the internal part of the periphery, diminishing the pulp-chamber in a progressive way.

This calcification becomes generally complete about the age of thirty-five or forty years.

The two ways of nutrition, external and internal, must progress simultaneously until the calcification of the tooth is complete, and once fully completed, one ought to suppress it, in order that it shall not hinder the function of the other one. When one of the functions ceases to be a physiological one, it becomes fatally pathological, constituting in this way a real danger. It is therefore necessary to insure the integrity of the first function by suppressing the second, which has become harmful, and so preventing in this way a reaction upon the point that we desire to preserve physiologically.

In order to delay the senile loss of the dental organs, we have had recourse to the following means of operation, which is very distinct from any other:

The results produced by our trephining show how to suppress a mode of nutrition which has become useless, which, should it have continued to progress, would infallibly have brought a case of anticipated senility favoring the loss of the tooth.

When one calcification is complete, and fully and duly constituted, in spite of the semiological interest, it is advantageous to perform our operation, by trephining the crown of the tooth through the axis of the central pulp-chamber. With a small drill perforate the teeth which have a single root, using a larger one for teeth which have more than one root, carefully removing the enamel at the spot where the drill acts—best done by the use of drills made from small chips of diamond, which act rapidly on the surface of the tooth. Extract the dental pulp by the use of the nerve broach, which is used after obtunding the tissue with cocainephenate, or, if the root-canal is mechanically inaccessible, by the use of the electro-cautery. Fill the canal or canals, the pulp-chamber, and the trephined part of the crown by any of the different methods of filling as commonly used. It is in these cavities, perfectly cylindrical in shape, that one can employ as a means of closure, small pegs of compressed wood, and this wood must be antisepticized.

In order to realize the most complete result, one ought to practice the operation before the pathological state of the tooth is very advanced. In fact, if one waits till the inflammation, succeeding the irritation of the pulp, has become chronic, to the point of damaging the peridental ligament, with the gathering of calculus, becomes injurious and brings about alveolar alteration, the operation of trephining will infallibly prove useless, if not dangerous, as the tissue being injured, cannot stand similar transformation. In such a case it will be necessary to employ the process of resection, between the alveolaris, adopted by Messrs. Martin and Poinsot, or even by the replacing method recognized by Messrs. Magitot, David, Castinel, etc.

If the typical condition be a little modified by pathological state, it can be discovered in its primitive stage by a careful examination, or by the electric light, permitting the examination of the dental tissue through its transparency.

The meeting then adjourned, to meet at 2:30 P. M., Friday.

### SECTION 5.—DENTAL AND ORAL SURGERY.

Dr. Brophy in the chair.

A paper was read by Dr. Louis Ottofy, Chicago, as follows: "History and the Present Status of the Transplantation of Dental Tissues."

The transplantation of dental tissues in the oral cavity is practically confined to the teeth themselves, and hence this paper will treat of that subject alone. Transplantation of teeth has been practiced for several centuries; it has, however, remained for those of the latter part of the nineteenth century to devise scientific means for the proper performance of these operations.

It seems almost impossible that successful transplantation could have been practiced without antiseptic precautions.

In transplantation we include the subject of implantation.

The history of tooth transplantation includes the undisputed claim of John Hunter to its introduction, a quotation from the proceedings of the Central Association of Dentists, Heidelburg, in August, 1881, in which M. Witzel submitted casts of a case where he had replanted a diseased incisor and implanted a dead incisor, for a lady fifty years of age.

Dr. Ottofy also quoted Dr. Bogue as having learned in Paris, several years previous to 1885, of several cases of the implantation of teeth into artificially prepared sockets. These teeth after becoming firm, and remaining so for over a year, gradually loosened from absorption of the roots.

He continued: As is well known, it was some years subsequent to this that Dr. Wm. J. Younger, of San Francisco, Cal., re-introduced the subject to the profession of the United States. So that here, too, we have, if you please, the Vikings followed by Columbus. To whom credit, and in what measure it is due, is for you, gentlemen, to say.

Regarding the present status of the subject, he said: The operation has received but limited recognition in the permanent literature of the last few years, and reference to it is made more in the spirit, as if the operation were merely a curious experimental novelty. My observation and experience in implantation leads me to express the following statements:

1. Implantation has been practiced for a sufficiently long period to entitle it to be accepted as a legitimate operation in oral surgery, in regard to which the dental surgeon should have the privilege to perform, assuming the attendant risks as he is doing in other operations. This statement is made as the result of observations extending over a period of nearly eight years.

2. That in view of the fact that all dental operations are more or less of a transitory character, implantation deserves to be classed as a permanent operation, in the same sense in which other operations performed by dentists are considered permanent, and that it should not be considered merely a fanciful experiment.

3. That no operation of the dentist so nearly reproduces nature as that of an implanted tooth, and the result, when successful, in appearance and comfort cannot be excelled by any other operation within the domain of the entire science of dentistry.

4. That the operation should not be performed in any cases, except those which have been as carefully selected as they would be, and as they are, for other operations.

It is not within the province of this paper to describe the modes of operation and the various details, but to have the subject considered by this representative international body, in order that this branch of oral surgery may receive its proper recognition.

### DISCUSSION.

In the discussion of this subject Dr. Lester gave the history of an implanted tooth, in his own mouth, which did good service for two and a half years, and said: If I had been careful of the tooth it might have been serviceable to-day. I was constantly biting hard substances to test it, and at the end of the time stated, the tooth loosened and was lost as temporary teeth are.

He said: I have, myself, implanted quite a number of teeth, and if it were not for the absorption of the root of the tooth implanted, the operation would be, in my opinion, a success in many cases. Many patients desire the operation if they can be assured of success. The tendency of loss, by absorption of the root, is so great that, unless it can be overcome, I think there is no future for implantations.

Dr. Younger said: I recently heard from a lady, in Paris, for whom I implanted five teeth, four bicuspids and one molar, over eight years ago, and she reports that they are now perfect. Also, another case, that of her son, over five years ago, and in each case, no one would know from examination but what they were teeth that were indigenous to the mouth.

Dr. Barrett: In implanting teeth where there is an absorption of the alveolus, do you get a development of the alveolus around the tooth?

Dr. Younger: Yes, sir; it is done within two or three months. So we have a deposit of the alveolus, the same as where the tooth had grown in the first place.

The question was asked: Does the pericementum become re-juvenated?

Dr. Younger: The cicatricial tissue forms a pericementum around the tooth.

The question was asked: What has been the condition of the pericementum upon extractions where they have been failures?

Dr. Younger: One case I reported; I had implanted a tooth for a lady, and the next morning I discovered a fissure, so I removed the tooth, after being in only one night, and I laid it by. Three years afterward a gentleman came in from San Francisco, who had lost three teeth during the war by the explosion of a cannon, and this tooth was the only one I had to match his. The patient said he did not care for the beauty, but for the utility of the tooth. So I implanted three teeth, the cracked tooth being one of the three. About two and a half years after that the tooth split. One portion was firmly fixed in the alveolus, and the other was loose, and yet gave him pain when he tried to pull it off. I tied the teeth together and tried to get the rest of that tooth out, and, knowing how hard they are to get out, I took a very high hold, and extracted the portion that had become attached, and that portion seemed to be perfectly covered with healthy pericementum. I immediately put it in cold water, and sent for Dr. Jacobs, who was recommended to me. He had read a paper before the local society, saying there could not possibly be any vitality in the tooth after it had been extracted, and I thought I would rather have it in the hands of an enemy than a friend, so I sent for him, and he said: "If that is what it seems to be, I will have to chew my words." He came back and said that he had found it to be pericementum, and that the tissues underneath were healthy tissues.

Dr. Bentley: How do you account for the absorption of some of these roots?

Dr. Younger: I only know they do absorb.

In reply to another question, Dr. Younger said: I calculate upon about twenty per cent. of unsuceessful implantations. The first operations of which I took a record were thirty-six, and out of those I am not able to find more than nine that have been failures, and those have been in eight or more years.

In regard to the reproduction of pericementum Dr. Barrett said: We find interspersed throughout the tissue of the bone, the osteoblast, and wherever that osteoblast exists, there may be a reproduction of the bone-tissue. Now he makes in his operation an artificial cavity in the bone itself. What is the consequence, the reproduction of periosteum or pericementum? I conceive that there is a reproduction of periosteum or pericementum, but that there shall be any persistence of life of the periosteum after the tooth has been entirely desiccated seems to be utterly impossible.

We are all under great obligations to Dr. Younger for the persistency with which he has urged this, until it has, as I believe, come to be an established system of practice. It has come to stay, and it is not a matter of experiment hereafter. That teeth are ocasionally lost is a matter of course, but do you never make a second plate? If a tooth is lost, as it occasionally will be through some malnutrition of the system, implant another just as you would put in another artificial tooth. It is not a failure of the operation, either from a surgical or physiological standpoint.

Dr. Whitney, Hawaii, related a case in which he had implanted a bicuspid. Six years later the lady came to his office in great distress. She said, "Doctor, something has happened to that tooth." Dr. Whitney said, I looked at it, and saw it had been split from top to bottom. In attempting to remove the split portion, I found it very firmly attached, as any live tooth I had ever found that had been split off from a section of the tooth. I attempted to extract it with my fingers, but could not. I then drew a thread around it, and afterwards a ferrule, and it remains there to-day. Now the question is, what held that? Was it a living con nection? I examined it, and could not see that there was any difference between that and any other living tooth that I had ever examined.

Dr. Younger: I think there is no proof of vitality like sensation. I have known of an implanted tooth upon which was a little tartar, and on touching it, found it hurt. So I pretended to touch another tooth, and put the point on that same one, and that tooth was as sensitive as any other tooth that had grown in the patient's mouth.

Dr. Younger then explained his method of using ligatures, after which the Section adjourned to meet Friday, August 18, at 2:30 P. M.

SECTION 6.—OPERATIVE DENTISTRY.

The meeting was called to order at 2:30 P. M., by Dr. Jarvie.

Dr. Geo. W. Whitefield, of Evanston, Ill., read a paper on "Soft Teeth and Galvanic Action between Gold and Baser Metals."

After a brief discussion of electricity, its forms, methods of production, and modes of action, Dr. Whitefield said: A battery may be formed by dissimilar metals in the mouth, where the fluids of the mouth are the exciting media, in which gold will be the negative element, as the fluids of the mouth have no action on this metal. The positive element is the basic metals, whether used separately, as tin, or, as most commonly, the combination of mercury with tin, silver, zinc, and copper, as in the amalgams in use.

With gold and tin to form the voltaic pair, the base metal soon becomes coated and the current practically ceases, but, with amalgam, the mercury performs the same office in the mouth that it does in the laboratory. The action will vary as the conditions are changed; naturally where food is left between the teeth to decompose, the acid resulting from such fermentation will form a more exciting media than normal alkaline saliva. Where a filling is left rough and jagged, overhanging and irritating the gums, it presents an exaggerated surface to be acted upon; besides, by irritating the gums, it causes a secretion from them that forms an excellent, exciting fluid. This is unfortunately the too frequent result of careless operating. If gold and amalgam touch in the same tooth there is practically less destruction around the gold filling. The galvanic action is so violent that the surface of the amalgam filling is soon destroyed; leaving the silver, gold, or platinum, of which it is composed, on the surface, which practically changes the amalgam to nearly a silver surface, thus raising it to nearly a negative metal, while, besides this, the coating protects even the surface of the silver. With regard to very old plugs of amalgam, their surface is no longer amalgam, it is negative metal.

Galvanic action has a tendency to accelerate the blood-flow, producing hyperemia and hyperesthesia, and in some cases violent nervous phenomena. This is especially the case where, from the situation of the fillings, the energy is accumulated, then suddenly discharged, producing shock, as any one can demonstrate by touching a bit of zinc or a blade of a penknife to a filling in his own tooth. The current is generated from all parts of the elements that come in contact with moisture, and are not protected by a coating.

As gold remains bright on all its surfaces, and as moisture pervades the whole tooth, no matter how well the filling is inserted, moisture will reach it by way of the intertubular spaces, consequently electrolytic action can take place from all portions of the gold element, naturally causing considerable destruction around the gold filling.

In the laboratory of the Northwestern University, in Evanston, Professor Crew tested to ascertain the voltage of a battery composed of a small gold and a small amalgam filling, the fillings being held in the mouth so that the saliva should be the exciting fluid. The battery yielded .9 of a volt, equal to a Daniell cell.

Among the commonest elements found in the mouth is chloride of sodium (salt); galvanic action readily breaks up this compound, the chloride liberates oxygen and unites with the hydrogen of the water, forming hydrochloric acid. Other acids may be formed in this way. The electrolytic action and the acids thus formed are sufficient to roughen the surface of the teeth to give lodgment to colonies of microbes.

Chlorine in its nascent state will readily unite with the mercury of the amalgam, and the chlorides of mercury may be formed in sufficient quantities to produce symptoms of mercurial poisoning in those susceptible to its influences. These salts of mercury may also explain the immunity from decay of teeth stopped with ill-fitting amalgam plugs, the germicidal effects of the mercury being sufficient to prevent colonization by micro-organisms, and consequent destruction of the tooth.

The oxygen might, from peculiarities of the individual case, unite with other salts than those of the teeth, and the same with acids.

The usual result of placing amalgam in the back teeth, while gold is placed in the front teeth of children, will explain why the gold fillings have to be renewed so often; also, that in the electrical action is a clue to the oft-repeated tale of patients, that amalgam stands better than gold, as the amalgam still remains and the gold fillings, that have been replaced several times, are loose again.

If amalgam must be used, use those grades that will readily become coated, as the coating will reduce the galvanic action, protecting the plug from the fluids of the mouth, except where attrition and brushing keep them bright.

Fortunately, we have other than metallic filling-materials—cements and gutta-percha. Often in practice when called upon to do what is best for the teeth, where it seems almost beyond belief that the pulp's vitality can be retained, yet by thoroughly preparing the margins of the cavity, sterilizing the decay covering the pulp and filling with cement, we find that in one to three years the disintegrated dentine is easily scraped from that which has hardened by the treatment of sealing up the ends of the tubuli, and we have an ideal condition—dense, hard dentine.

### DISCUSSION.

In the consideration of this paper, Dr. J. Y. Crawford said: It is my firm conviction that it is the duty of the dental profession to denounce three evils: One is, to perform the operation of extracting teeth painlessly; another is, the use of methods which develop galvanic action in the treatment of teeth; and the third is, the use of amalgam in the treatment of dental caries. I believe the aggregate results following the use of the baser metals have been more hurtful than beneficial to the human family. I was told a good many years ago that I could not practice dentistry without the use of amalgam. I say, to-day, that this statement is false, because I do so practice it more successfully than when I had the nefarious stuff in my office.

Dr. Pruyn: This is a question upon which I have experimented constantly, and if the experience of twenty years in the use of amalgam is worth anything, I would like to add my testimony for the proper use of amalgam. The curses that have been heaped upon amalgam had better been heaped upon the careless operators. You have considered it a cheap material, and you carelessly prepare your cavities when you use amalgam fillings. If you prepare your cavity as you would for gold, of course, you will have failures; amalgam can never be placed in a cavity that has an attenuated edge. You must have good borders, so there will be no chance for the overlapping edges to break down. I never have seen the alarming results from electricity generated in the mouth, that the essayist has mentioned to-day. The combination of gold and amalgam as a filling-material will preserve the teeth better than gold alone, better than amalgam alone.

After brief closing remarks, in reply, by Dr. Whitefield, the subject was passed and Dr. Jarvie, the Chairman, requested that Dr. Gordon White, of Nashvile, Tenn., explain his operation of sponge-grafting.

Dr. Gordon White: At the request of the Chairman, I make what is practically my third report on the "Treatment of Chronic Abscesses caused by Diseased Roots." I have had quite a good deal of experience in this character of operation. We are familiar with the treatment by removing the apex of the root with an ordinary bur. Very frequently the disease extends from one to two-thirds of the root from the apex. A great many of us will amputate the root, and a great many will leave the cavity in the bone to take care of itself. Several years ago, I began the treatment of filling with sterilized sponge this cavity, after having burred out the diseased bone. I have quite a number of these cases which have proved very successful. Of course, every instrument is sterilized, I simply fill the cavity with a little piece of sponge, and find I have no further trouble.

For sterilization I depend upon water that has been boiled; I use water at about 160 to 180 degrees. Just dip your instrument in, and wash it with water that has been sterilized by boiling. I dip the instrument in chloroform, which aids somewhat in the sterilization. I then dip it in carbolic acid and proceed with the operation. I cut off the end of the root with an enamel-bur that is furnished by Ash & Co. I frequently amputate the entire root of the molars. I have quite a number of lower molars that are doing excellent service, after the entire distal root has been removed at the bifurcation, as a surgeon would take off your finger at the joint. I sterilize the sponge by keeping it in a solution of two grains to the ounce of bichloride of mercury, at a degree of one hundred and sixty-four, for perhaps half an hour. I got my first ideas of this operation from our grand old friend, Dr. Atkinson. I have since been practicing it pretty generally, and my friends in the South know of quite a number of my successful cases. I just dismissed a case a few day before I left home, where I amputated the buccal root of the first superior bicuspid, and filled the cavity with sponge. The patient came in a few days before I left, and the wound was entirely well. For chronic fistulous openings, where the roots have been filled, I treat them that way almost invariably, particularly if the necrosis is extensive. When the wound is well, the tooth is almost as tight as its neighbor.

A member: What becomes of your sponge?

Dr. White: It is absorbed. The gum will heal entirely over; there may be, however, the slightest mark after the operation. Of course, the pulp-canal is always filled before the operation is performed.

Dr. George Cunningham, Cambridge, England: In discussing this question, the new point is the use of sterilized sponge for filling these cavities. I am happy to say it has been a good many years since I learned the operation of the amputation of roots; I am also glad to say that I do not have occasion to have recourse to it. I am not prepared to announce any objection to the sterilized sponge, because I use the sterilized sponge in another way. The sterilized sponge has been used for the purpose of producing artificial dentine. I should say that the sterilized sponge might give very satisfactory results, indeed; at the same time, I am not quite clear as to its advantages over the other method. The difficulty I have is to find out where the end of the root is.

Subject passed, and the meeting adjourned to 2:30 P. M. Friday.

SECTION 7.—PROSTHESIS AND ORTHODONTIA.

The Section was called to order by the Chairman, Dr. Goddard, at 2:30.

Dr. C. S. Case read the following paper: "Some Principles Governing the Development of Facial Contours in the Practice of Orthodontia."

The writer stated his belief that we are at the beginning of a renaissance in orthodontia which will not be satisfied with the mere correction of malposed teeth, but will include as indispensable the correction of all facial deformities, which have resulted from irregularities of the teeth and jaws, and the development of every esthetic contour of the face that can be accomplished by a scientific application of force to the underlying bony structure.

In examining dental literature in this department, one is surprised to find so little said in regard to the movement of the roots of teeth, and the methods by which it may be accomplished; and in no place have I been able to find a single proposition for the outward or inward movement of the roots accompanied by a relatively slight change in the occluding position of crowns, and nothing in regard to the movements of the roots, for the purpose of giving a more perfect contour to the face by changing the shape of the underlying bone.

He said, the purpose of the paper was to show how force may be applied to the outward and inward, as well as to the lateral movement of the roots of the teeth; and to illustrate also the importance of this possibility, when it is observed in this operation that the bones of youth do not remain stationary to be plowed through by the roots in a process of retrogressive metamorphosis, but that a considerable portion of the bone in which the teeth are imbedded is carried with the roots in proportion as they are changed in position, thus enabling one to regulate many imperfections of the face, by changing the shape and surface contour of the frame which supports and gives character to the features over all that portion which can be affected by a movement of the bones contiguous to the roots of the teeth.

The facility with which the entire inter-maxillary process can be carried backward or forward, under a proper application of force to the incisor teeth, has been a source of surprise and pleasure to me from the time when I first attempted the operation—less than a year ago.

The essayist then described numerous deformities of the lower portion of the face, due to depressed or protruded roots of teeth, of which the crowns have comparatively perfect alignment and occlusion. In other cases, to give proper external contour to the face requires that the entire dental arch and alveolus should be expanded, and the force so applied and controlled as to retain the teeth in an upright position, especially in the process of carrying the anterior teeth forward.

An abnormally prominent chin; protrusion of the upper lip when it surges into the nasal septum; depressed, anterior, inferior teeth; and other inartistic relations of the features governed by the position of the teeth were defined; and methods of procedure indicated.

If by force appliances attached to the crowns of the teeth of young persons, the roots and the alveolus can be forced outward or inward to any desired extent, a new field will be opened to the practitioner in orthodontia, a principal feature of which will be the correction of many deformities to the face, heretofore considered beyond the reach of orthopedic surgery. If the crowns of the teeth only are forced backward or forward, the facial defect is only partially remedied and the real deformity far from being removed—if not increased, as it may be, by the tendency of the roots to tip in an opposite direction. But if, on the other hand, the teeth are firmly grasped by appliances so constructed that the force can be applied directly to the roots while the antagonizing ends of the crowns are fixed or controlled in their movement, it will be found that the roots as well as the immediately surrounding bone will be moved, and made to take a position which will give a far more pleasing appearance to the face.

The apparatus devised by him for "Facial Contouring," in various conditions, was minutely described, of which an abstract cannot be given.

At the conclusion of the reading Dr. Case presented a number of models of cases showing the action of the contouring apparatus when put into practical use.

DISCUSSION.

Dr. G. V. Black, Jacksonville, Ill., said: All of you who are acquainted personally with orthopedic operations know how easy it is to effect the bending of the long bones of the human frame. The bones of all parts of the human body in children are so soft that they may be easily bent to almost any extent by persistent effort, the bones of the jaw as readily as others.

Dr. Mitchell, London, asked at what age the best results could be hoped for from operation, whether it was not best to operate before fifteen years of age?

Dr. Case replied: The earlier the better, after attachment could be made to a permanent tooth.

Dr. E. A. Bogue, New York, said he wished to thank Dr. Case for devising the appliance shown this afternoon. He had never before seen anything as good. Cases of prognathous upper jaws are the hardest to treat, because, as in the case of Archimedes, we have had no place to put our fulcrum. Perhaps, now we will have found the place.

Dr. Goddard said: It has been acknowledged by almost every one that they have not succeeded in moving the roots of the teeth. I think the appliance shown to-day will do it if anything will.

The Section next took up the discussion of the topic of the day: "What Are the Etiological Factors in the Production of (a) the Protruded Lower Jaw; (b) The Retracted Lower Jaw?

When this Form of Irregularity Is Corrected by 'Jumping the Bite,'. Does a Compensating Adjustment Take Place in the Temporo-Maxillary Articulation?"

Dr. E. S. Talbot, Chicago: In regard to the protrusion of the lower jaw there are two factors, outside of dentistry, which enter into the causes: Heredity and excessive or tardy development on account of a neurotic state. Just what produces this neurotic state we do not know. It may be mixture of race, intermarriage among relatives, use of alcoholic liquors, or disease. If we examine the skulls of people of several centuries ago, or those of some few races existing to-day, we will find the jaws generally normal. But the advance of civilization has produced two types among our people, one of over-stimulation, or over-developed brain, and a degenerated or under-developed brain. In either case we are liable to have an unstable development of the osseous system and other organs of the body. I have noticed such deformities, in from forty-five to sixty-five per cent. of people of average development. The jaws develop in accordance with development of the brain. We get from this, at times, protrusion, and at other times arrest of development of the lower jaw.

Dr. Newkirk thought the cause was more frequently the irregularity of the teeth, but as to why one person has a prominent lower jaw and another a prominent upper jaw, I do not think any one can tell, nor do I think we can tell why we differ in other ways. The question is not practicable.

Dr. Talbot said: For several years he has been studying the causes of irregularities, and was able to correct them with very simple appliances in some cases, because of the knowledge gained. He asked the opinion of those present as to the possibility of "jumping the bite" the distance of one or two cusps, by moving the jaw forward so as to make a new joint.

Dr. Goddard: Did not think there was a possibility of "jumping the bite" permanently.

Dr. W. N. Morrison described a case in his practice and the Section then adjourned to Friday.

### SECTION 8.—EDUCATION, LEGISLATION AND LITERATURE.

The Section was called to order by Dr. J. J. R. Patrick, Chairman, at 2:30 P. M.

Dr. Wm. O. Kulp, Davenport, Iowa, read a paper entitled, "Dental Nomenclature," which presented an amplification of the system of terminology he had advocated before the American Dental Association in 1885.

Dr. J. L. Secher, delegate from Copenhagen, Denmark, explained a system of notation for the teeth and cavities which is very simple, but deficient in that it makes no provision for locating fissures or the portion of a surface on which a cavity may be located. Dr. Secher's command of the English language being very limited, his remarks were confined to illustrations by figures, which, as stated by Dr. Kulp, was a revival of the system of the elder Zsigmondy, given to the profession in 1861.

### DISCUSSION.

Dr. A. C. Hewitt said: As we are particularly an English-speaking people, he wished to ask if, in case the Congress adopts a system of nomenclature it would be construed as international in its scope, or would it simply be one to be adopted by the English-speaking people of America?

Dr. J. J. R. Patrick said: There never would be formulated a universal system. These papers were only the expression of individual opinion. No discretion was given the Sections. The papers were accepted, read, discussed and handed to the Publication Committee.

Dr. Hewitt, expressed his hearty approval of the system proposed by Dr. Kulp, which appears to be simple, accurate and complete, and complimented the acute, nice distinction in the use of words. We want English words with which to propound English questions, to be answered by English-speaking pupils.

No remarks were offered on the paper of Dr. Whitney, on Hawaiian skulls, referred to this Section.

Dr. H. B. Noble, Washington, D. C., read a paper on "Dental Legislation."

There seems to be a surprising lack of correct information by those who talk and write upon dental legislation, and many of our dental laws have been based upon false representations of what was required of other professions.

Many of the dental laws have not been as carefully drawn by a good lawyer as they should have been.

There is a feeling that no professor in a college should be on an examining board. These are the very men best qualified to properly be on the board, and if we could have at least one teacher on every board it would be a good thing, and tend to bring into harmonious relation our colleges and examining boards, for they ought to work and fit together in their work, which has the same end and aim in view—the education of dentists.

Let us guard well the entrance to the dental field, and see that no unworthy man is allowed to pass in, but let us not undertake to divide the field into sections, allowing a man to practice only in his section, and refusing to allow him to pass from one field to another without facing an examining board, whose qualifications are unlikely to be superior, if, indeed, they equal those of our college teachers and professors. Our colleges are a source of strength, upon which we must rely for educating and elevating our profession, and all our laws should be directed to the encouragement of college education and systematic training.

This paper was passed without discussion, and the Section adjourned to 2:30 Friday.

[*To be continued.*]

